# The Use of Wearable Activity Trackers Among Older Adults: Focus Group Study of Tracker Perceptions, Motivators, and Barriers in the Maintenance Stage of Behavior Change

**DOI:** 10.2196/mhealth.9832

**Published:** 2019-04-05

**Authors:** Anastasia Kononova, Lin Li, Kendra Kamp, Marie Bowen, RV Rikard, Shelia Cotten, Wei Peng

**Affiliations:** 1 Department of Advertising and Public Relations Michigan State University East Lansing, MI United States; 2 Department of Media and Information Michigan State University East Lansing, MI United States; 3 Department of Biobehavioral Nursing and Health Informatics University of Washington Seattle, WA United States; 4 Center for Innovation and Research Michigan State University East Lansing, MI United States

**Keywords:** aging, wearable electronic devices, biobehavioral sciences, transtheoretical model of behavior change, exercise, physical activity

## Abstract

**Background:**

Wearable activity trackers offer the opportunity to increase physical activity through continuous monitoring. Viewing tracker use as a beneficial health behavior, we explored the factors that facilitate and hinder long-term activity tracker use, applying the transtheoretical model of behavior change with the focus on the maintenance stage and relapse.

**Objective:**

The aim of this study was to investigate older adults’ perceptions and uses of activity trackers at different points of use: from nonuse and short-term use to long-term use and abandoned use to determine the factors to maintain tracker use and prevent users from discontinuing tracker usage.

**Methods:**

Data for the research come from 10 focus groups. Of them, 4 focus groups included participants who had never used activity trackers (n=17). These focus groups included an activity tracker trial. The other 6 focus groups (without the activity tracker trial) were conducted with short-term (n=9), long-term (n=11), and former tracker users (n=11; 2 focus groups per user type).

**Results:**

The results revealed that older adults in different tracker use stages liked and wished for different tracker features, with long-term users (users in the maintenance stage) being the most diverse and sophisticated users of the technology. Long-term users had developed a habit of tracker use whereas other participants made an effort to employ various encouragement strategies to ensure behavior maintenance. Social support through collaboration was the primary motivator for long-term users to maintain activity tracker use. Short-term and former users focused on competition, and nonusers engaged in vicarious tracker use experiences. Former users, or those who relapsed by abandoning their trackers, indicated that activity tracker use was fueled by curiosity in quantifying daily physical activity rather than the desire to increase physical activity. Long-term users saw a greater range of pros in activity tracker use whereas others focused on the cons of this behavior.

**Conclusions:**

The results suggest that activity trackers may be an effective technology to encourage physical activity among older adults, especially those who have never tried it. However, initial positive response to tracker use does not guarantee tracker use maintenance. Maintenance depends on recognizing the long-term benefits of tracker use, social support, and internal motivation. Nonadoption and relapse may occur because of technology’s limitations and gaining awareness of one’s physical activity without changing the physical activity level itself.

## Introduction

### Trackers Are Beneficial to Older Adults, Yet, Underused

The focus of this study was on exploring the nuances of maintaining the use of wearable activity trackers and reasons to discontinue activity tracker use in the population of adults who are aged 65 years or older. Academic and industry research has shown that the use of activity trackers can increase physical activity through continuous monitoring of activity progress, motivational messages, social support, and many other empirically tested behavioral change techniques [1-4]. Activity trackers facilitate physical activity, which is beneficial for older adults because of the protective power of physical activity against diseases associated with older ages (eg, heart rate) [5].

Despite the evident benefits of activity trackers for older generations, digital care today is more available to younger populations, leaving older adults on the periphery of the industry [6]. As little as 7% of older adults owned an activity tracker in 2014 [7]. Although many adults are now aware of this technology with its increased popularity, this population still shows slow rates of adoption that depends on many factors, including activity tracker trial and price [8,9]. Only a few studies have been done to understand how and why older adults maintain the use of activity trackers and why they choose not to use or stop using this wearable technology [10-12]. Even if individuals decide to use activity trackers or are given a tracker at no cost, it does not guarantee that they will continue using them on a long-term basis. Overall, 1 in 3 activity tracker users of all adult ages stop using the device within 6 months after purchase [7,13]. The length of use correlates with age, where adults who are aged 70 years or older quit using activity trackers in only 2 weeks [14].

Little comprehensive evidence exists with regard to the long-term use of activity trackers by older adults [10,11]; motivations for long-term use [2,3]; and differences between nonuse and short- and long-term use. To study how older adults maintain the use of activity trackers and why their motivation to maintain activity tracker use grows stronger or fades over time, we applied the transtheoretical model (TTM) of behavior change, with a focus on the maintenance of desirable health behavior and relapse (discontinued activity tracker use). In the literature review below, we first introduce the theoretical framework of TTM, then discuss physical activity and activity tracker use in older adults, and finish by addressing previous research on the maintenance of activity tracker use behaviors.

### Transtheoretical Model of Behavior Change

This study explored the motivations and barriers associated with the sustained use of activity trackers through the lens of the TTM. The TTM employs the stages of change to integrate processes and components of change across major theories of intervention [15-17]. A key element to the TTM is the stage of change as the construct is the temporal dimension to the framework. The TTM proposes that change is a process that unfolds over time and progresses through a series of 6 stages. In the precontemplation stage, people do not plan to change their behavior in the near term because they are either uninformed or underinformed about the consequences of their behavior. For example, older adults might not be aware of the existence of activity trackers or realize what health benefits activity tracker use entails. The costs and beneﬁts of change potentially produce uncertainty, and some people remain in contemplation for extended periods of time. For instance, activity tracker use could be perceived as beneficial, but the price and lack of technology skills could stop older adults from adopting it. People begin to take signiﬁcant steps toward the behavior change in the preparation stage. There is a plan of action and a critical stage for recruitment into action-oriented programs. In the action stage, an individual makes specific evident lifestyle modiﬁcations, such as wearing an activity tracker every day and changing daily routines to increase trackable activities.

The continuation of the specific behavior and lack of relapse is the maintenance stage. Relapse, however, is a common occurrence in the maintenance stage where individuals abandon new behaviors and return to old ones (former tracker users in this study). Older adults may successfully maintain the use of an activity tracker for a period of time; however, some may stop at some point. What makes older adults continue to use the technology and what contributes to its abandonment is the focus of this study. During the maintenance stage, individuals have made specific and apparent changes to their lifestyles, and their continuous efforts to prevent against relapse no longer require frequent applications of the change processes as one would during the action stage. Individuals in the maintenance stage (long-term activity tracker users in this study) are less likely to be tempted to revert to previous behaviors, and they have increased confidence and self-efficacy in keeping up with the changes. When making a behavior-related decision, individuals in the maintenance stage are more likely to consider and be influenced by the pros rather than the cons associated with the behavior [18]. The estimated duration of the maintenance stage is about 6 months to 5 years before the termination stage [17].

### Wearable Activity Trackers Increase Physical Activity

Activity trackers refer to sensor-based wearable devices that automatically track and monitor various indicators of physical activity, such as steps taken, stairs climbed, duration and quality of sleep, pulse or heart rate, calories consumed or burned, and even mood [19]. Activity trackers synchronize these data with users’ personal accounts, ensuring easy access from any device. Although the specific features available depend on tracker brands and models, older adults typically use activity trackers to monitor the distance covered, steps taken, calories burned, sleep time, and heart rate [11], making activity trackers a convenient tool for this age group that provides feedback about physical activity amount and intensity.

Physical activity in older adults reduces the risk of chronic diseases, such as cardiovascular disease, stroke, obesity, and hypertension; improves cognitive and mental health; lowers the chance of falls; and helps maintain a longer independent life [20-22]. The minimum recommended level for older adults is 150 min of moderate-to-vigorous physical activity per week [23]. Despite this recommendation, older adults constitute the most sedentary age group [24-26]. Almost 84% of older adults aged 65 years and older do not meet the aerobic and muscle-strengthening physical activity requirements [27], which makes activity trackers a particularly relevant technology for this age group. In particular, physical activity recommendations for older adults focus on moderate-intensity aerobic and muscle-strengthening activities such as walking, jogging, bicycle riding, yard work, and gardening [22,28]. Some of these activities are tracked by wearable technology.

Activity trackers have the advantage of boosting physical activity through the integration of empirically tested behavioral change techniques such as goal setting, self-monitoring, social support, social comparison, feedback, and rewards [3,4], in contrast to antecedent technologies, such as pedometers. Self-monitoring and goal setting have been especially effective in promoting self-efficacy and physical activity in interventions [29]. It has been found that even though wearing a new piece of health technology is a novel activity for older adults, they appreciate the activity tracker’s contribution to self-awareness and goal setting. Activity trackers provide older adults with relatively unbiased data about basic activities. In addition, older adults view activity trackers as helpful motivators in achieving walking goals and competing with themselves [8]. Another important advantage of activity tracker use in the *65+* population is social connection. For a population that is characterized by social isolation and loneliness [30], technology that addresses social connectedness needs is perceived as helpful in overcoming barriers to increase physical activity [31].

Adults in general and, specifically, older adults who started using wearables have been shown to increase daily activity levels [1,10]. A 7-month study of 18 participants (aged 36 to 73 years) who were given a wearable tracker [32] found that 16 participants continued to use it after 7 months. The benefits of use included weight loss, social connection, and increased activity awareness. Participants aged 60 years and older who were given a tracker reduced waist circumference and increased step count during another 12-week study [2]. African American and Hispanic older female participants, who tested a newly developed tracking device in a 7-week study, increased their physical activity level, lost weight, and lowered blood pressure levels [33]. Activity trackers have been found to be more effective than their *predecessors*, where sedentary female older adults who used digital trackers significantly increased their physical activity compared with those who used pedometers [1]. A tracker that delivered prompts via short message service has also been found effective in increasing moderate-to-vigorous physical activity among overweight and obese adults [34].

Although activity trackers can be helpful in increasing physical activity, this technology is not ideal. For example, in a study with 8 adults who were aged 75 years or older, 3 participants experienced technical problems with the activity trackers, preventing them from gathering any activity feedback [35]. Participants reported that they could only get the activity tracker to work 78% of the time.

### Activity Tracker Use Maintenance Among Older Adults

To date, there is a dearth of research employing the TTM to examine the efficacy of activity tracker–based interventions to maintain physical activity level, along with activity tracker use, among older adults [36,37]. Research based on a general population sample has shown that nearly three-fourth of the participants discontinued using activity trackers after 100 days from the initiation date, with most dropouts explained by technical failures and loss of activity trackers. In that study, 4 participants aged older than 65 years were among those who stopped using activity trackers within the first 100 days. Age, along with positive user experience, perceived activity tracker effects, and playing individual sports with family, positively predicted activity tracker use duration among those who continued using this technology after 100 days [38]. Another study with an older sample of patients recovering from a myocardial infarction (average age=56 years) found that activity tracker use was successfully maintained for over 1 year. Generally, participants used activity trackers several days a week but not on all days. Activity tracker use on all days was common only for the initiation period and only for a few patients [39].

### Study Objective and Research Questions

Although multiple TTM studies have investigated the adoption of health-related behaviors, fewer studies have examined behavior maintenance and its abandonment after a period of long use [16]. In this study, we focused on *activity tracker use* (not physical activity per se) as a beneficial health behavior and explored the factors that contribute to the successful maintenance of this behavior and, on the contrary, the failure to maintain it among older adults. We conducted focus groups with 4 types of activity tracker users who were aged 65 years or older: long-term, short-term, former users, and nonusers. Insights from long-term users helped us understand the strategies that they employed to maintain activity tracker use and activity tracker features that encouraged such activity tracker use sustainability. Talking with short-term users allowed us to compare activity tracker use strategies and perceptions at the initial and maintenance stages of tracker use. Experiences of nonusers and former tracker users were analyzed to examine whether and how activity trackers encouraged physical activity in older adults and what were the reasons for not using or abandoning this technology.

Research question (RQ) 1a: *What features and functions do adults who are 65 and older consider useful and would like to have on their activity trackers?*

RQ1b: *What are similarities and dissimilarities in perceptions of tracker features and functions between* *adults who are 65 and older* *at the maintenance stage of use (current long-term users), those who stopped using activity trackers (former users), those who were only at the initial stages of use (short-term users and non-users after the activity tracker trial), and those who are not familiar with the technology (non-users before the activity tracker trial)?*

RQ2a: *How do perceived benefits and motivators associated with older age drive activity tracker use in adults who are 65 and older?*

RQ2b: *How do adults who are 65 and older who are tracker long-term, short-term, former users, and non-users compare in terms of perceived benefits and motivators?*

RQ3a: *What barriers do adults who are 65 and older experience during tracker use?*

RQ3b: *What are similarities and differences in perceptions of tracker use barriers among* *adults who are 65 and older that are long-term, short-term, former users, and non-users of activity tracker* *?*

## Methods

### Recruitment, Participants, and Procedures

A total of 10 focus groups with adults aged 65 years and older were conducted. Furthermore, 4 activity tracker trial focus groups were conducted with tracker nonusers, where each participant attended 2 meetings. After the initial focus group, nonusers were offered an activity tracker to use for several weeks and then attended a follow-up focus group meeting. A total of 6 additional focus groups that did not involve an activity tracker trial were conducted with short- and long-term users and former users (2 focus groups were held per user type). Each focus group lasted approximately 2 hours. Each participant received US $20 for participation.

Up to 10 people were invited to each focus group with the use of convenience sampling strategies [40-42]. Participants were recruited through local senior centers, university’s listservs, and an online recruitment system at Michigan State University. The online recruitment system (Sona) provides access to over 7000 community-dwelling individuals who are interested in research participation (4% are aged 61 years or older). Flyers were distributed at local churches and senior centers, posted on Facebook networking groups, and published in a local newspaper. The general participant selection criterion was that participants had to be aged 65 years or older. Focus group locations included a local community center, hospital building, senior center, and university campus.

Older adults who were interested in our study contacted the research team through phone or email. A research team member provided a brief overview of the study and asked each participant screening questions regarding the use of activity trackers (eg, length of use) and date and time preferences for the focus group. Potential participants were invited to focus groups based on tracker usage. Individuals who had never used an activity tracker were invited to the tracker nonuser group. Short-term users were the current users who had been using a tracker for less than 6 months. Long-term users were the current users who had been using the technology for 6 months or longer. Former users were individuals who had used an activity tracker in the past but stopped using it.

The focus groups were conducted by the members of the research team who developed and refined the focus group protocol. The first focus groups were conducted by researchers with substantial experience in conducting focus groups and in-depth interviews. Those who were new to the procedure observed the focus groups first and then led their own under the supervision of more experienced colleagues. A structured guide was used for each focus group.

All materials and study procedures were approved by the institutional review board at the university where the study was conducted. Participants in each focus group were given time to read the consent form, which provided the option of not participating and withdrawing at any time. Participants were given an opportunity to not answer any of the questions asked. Data confidentiality was guaranteed. None of the participants refused to answer questions or stopped participation. Participants consented to audio and video recording by signing the consent form and a video release form.

After participants signed the consent form, focus group procedure began. Participants filled out a short survey to collect information about demographics, medical conditions or disabilities, baseline activity levels, and health status. Then, they were provided instructions about the focus group procedure (eg, there were no right and wrong answers and positive and negative opinions were equally important). The conversation started with an icebreaker question about favorite personal technology. After the tone of the focus group was set, they shared their associations with the term *wearable activity tracker*, described their experiences using it or observing others using it and provided ideas about an ideal activity tracker. Then the conversation moved to an in-depth discussion of reasons to start using the technology, or the lack of thereof, and motivations for continued activity tracker use. Benefits and barriers of activity tracker use and its influence on users’ lives were discussed as well. Former users also discussed reasons for abandoning the technology. At the end of each focus group, participants shared their opinions about the role of physical activity and wearable technology in maintaining one’s good health and were provided an opportunity to add anything else to the discussion. After the focus group was over, participants received compensation. Nonusers in the initial focus groups also received a tracker and instructions for its use.

#### Focus Groups With Activity Tracker Nonusers

Overall, 4 focus groups (2 initial and 2 follow-up focus groups) were conducted with older adults who had never used an activity tracker. The first set of focus groups ran in February (N1=10) and the second set ran in May (N2=7) to account for seasonal effects on physical activity [34]. At the end of the first focus group, participants were given an activity tracker, Garmin Vivofit 2, to use for 2 weeks in February and 4 weeks in May and then they returned for a follow-up focus group to discuss benefits and barriers to activity tracker use and acceptability of the technology. May focus group participants kept their activity trackers at the end of the follow-up focus group as part of the participation incentive. The 2 trials (February and May) differed because the suggestions from February focus group participants were incorporated into the May trial procedure. The suggestions included (1) making the trial period longer, (2) clarifying activity tracker use instructions and creating instructional and motivational videos, and (3) incentivizing participants by giving away an activity tracker as a gift (see Results for more details). Participants in the February group received a demonstration of how to put on the activity tracker, a brief overview of the features, and a written instruction book. Participants in the May group received everything from the February group plus instructional and motivational videos. The instructional videos demonstrated how to use the activity tracker (eg, how to put the tracker on and off, taking the tracker out of the wrist band, and how to use the tracker). Motivational videos were recorded with activity tracker long-term users (participants from the first long-term use focus group) who shared positive activity tracker use experiences and tips to maintain activity tracker use.

Garmin Vivofit 2 tracker is a wrist wearable device that records steps taken, calories burned, and distance traveled. The Vivofit 2 has a yearlong battery life and is water resistant up to 50 meters deep. As the Vivofit 2 does not need to be charged frequently, participants were expected to be less likely to forget the trackers on the chargers or stop wearing them. We also chose the waterproof option because of past evidence that water-related activities (eg, water aerobics) are especially popular among older adults [34]. The Vivofit 2 has a large display screen to ensure ease of reading for older adults.

#### Focus Groups With Activity Tracker Short-Term Users

A total of 2 focus groups (N3=2 and N4=7) were conducted with older adults who had started using activity trackers within 6 months before the focus groups (on average, they had used their activity trackers for less than 3 months). In addition, 4 of the short-term participants in the second focus group were the same participants who took part in the nonusers trial focus group in May. These participants kept their activity trackers and continued using them, which qualified them to participate in the short-term use focus groups. Garmin was the most used activity tracker in this group (n=5), followed by Fitbit (n=4).

#### Focus Groups With Activity Tracker Long-Term Users

A total of 2 focus groups (N5=7 and N6=4) were conducted with older adults who had been using a tracker for over 6 months at the time of focus groups. Participants had experience using either Fitbit (n=9) or Garmin (n=2). One participant had used Apple Watch in addition to her Fitbit. On average, they had used activity trackers longer than 12 months.

#### Focus Groups With Activity Tracker Former Users

Older adults who had previously used an activity tracker for any period of time but stopped using them before the focus groups occurred were invited to participate in 1 of 2 focus groups (N7=9 and N8=2). Participants predominantly had used Fitbit (n=7), but they also mentioned Garmin, Jawbone, Misfit, Nike, Gear Fit, manual pedometers, and special medical technology provided by a local hospital (eg, health management app). On average, their tracker use lasted for nearly 10 months.

### Data Coding and Analysis

Focus group conversations were audio recorded. Audio files were deidentified and transcribed using a Web-based service. Data were inductively analyzed; an exploratory thematic analysis was performed to indicate common nodes. A total of 5 coders iteratively analyzed the transcripts using NVivo, a software for qualitative data analysis. First, all coders analyzed 2 randomly selected transcripts. After the general agreement over the codes was established based on an in-depth discussion of each code [43], 2 coders analyzed 4 additional transcripts and another 2 coders worked with the remaining 4 transcripts. The fifth coder checked the quality of coding to ensure consistency. The coders met regularly until they finished coding all transcripts to discuss their interpretations of the existing codes, introduce new codes, reach mutual agreement on them, and create new code labels [43]. Some codes were aggregated into code categories during these meetings. When a disagreement was identified, it was discussed, and then the coders recoded the transcripts following the adjusted coding procedure. Thus, the coding rubric was being refined throughout the coding process because each new set of focus groups introduced new ideas and meanings. Intercoder agreement was ensured by extensive discussions of each new code [43]. At the end of the coding procedure, NVivo results from the coders of the same transcripts were compared to ensure that the coders agreed on the most prominent codes. Nodes were aggregated in more inclusive categories, which were used to derive themes. Multimedia Appendix 1 presents the map of the most prominent codes and code categories by focus group type. These codes were used to conduct thematic analysis and further understand differences among the 4 types of users. Quotations most representative of each theme were selected per agreement of all researchers from a larger pool of quotes that corresponded to each node and node category. Research team members reviewed multiple quotes and made suggestions about which ones to include in the article.

## Results

### Sample Description: Demographics and Beyond

Table 1 displays information about participants’ age, gender, race or ethnicity, and occupation by focus group type. Most of the focus group participants were female, especially in the long-term and former user groups. Participants in these groups were also younger. The majority of participants were white. In addition, 2 out of 3 short- and long-term users had completed graduate degrees, whereas about one-third of nonusers had only graduated high school and had some college education.

Information about chronic conditions, physical activity types and frequency, and activity tracker use length by focus group type is provided in Table 2. More short-term and former activity tracker users than other participants reported having a chronic condition. Former users showed a greater diversity of physical activity, whereas nonusers were mostly focused on walking. Walking and gardening were the most popular activities for long- and short-term users. Nonusers exhibited the lowest level of physical activity frequency.

**Table 1:** Participants’ demographic information.

Characteristic		Nonusers (n=17)	Short-term users (n=9)	Former users (n=11)	Long-term users (n=11)	All users (N=48)
Average age (years), mean (SD)		72.9 (7.5)	72.2 (9.9)	68.9 (2.5)	68.0 (3.1)	70.8 (6.7)
Age, range		66-94	66-94	67-73	65-73	65-94
**Race or ethnicity, n (%)**						
	White	15 (88)	7 (82)	11 (100)	9 (82)	42 (88)
	African American	0 (0)	2 (18)	0 (0)	2 (18)	4 (8)
	Hispanic	1 (6)	0 (0)	0 (0)	0 (0)	1 (2)
	Asian	1 (6)	0 (0)	0 (0)	0 (0)	1 (2)
**Gender, n (%)**						
	Male	7 (41)	2 (18)	2 (18)	2 (18)	13 (27)
	Female	10 (59)	7 (82)	9 (82)	9 (82)	35 (73)
**Education, n (%)**						
	High School	2 (12)	0 (0)	0 (0)	0 (0)	2 (4)
	Some College	4 (18)	2 (18)	2 (18)	2 (18)	10 (21)
	College	7 (41)	4 (46)	2 (18)	5 (46)	18 (38)
	Graduate	4 (24)	3 (36)	7 (64)	4 (36)	18 (38)

**Table 2:** Participants’ chronic conditions, physical activity levels, and activity tracker use length.

Health and physical activity descriptive		Nonusers (n=17)	Short-term users (n=9)	Former users (n=11)	Long-term users (n=11)	All users (N=48)
**Chronic conditions, n (%)**						
	Arthritis	8 (47)	3 (33)	8 (73)	6 (55)	25 (52)
	High blood pressure	7 (41)	6 (67)	6 (55)	3 (27)	22 (46)
	Obese	2 (12)	4 (44)	2 (18)	3 (27)	11 (23)
	Thyroid condition	3 (18)	3 (33)	2 (18)	2 (18)	10 (21)
	Heart disease	1 (6)	2 (22)	3 (27)	1 (9)	7 (15)
	Diabetes	1 (6)	4 (44)	2 (18)	0 (0)	7 (15)
**Physical activities, n (%)**						
	Biking	3 (18)	6 (68)	7 (64)	2 (18)	18 (38)
	Callisthenic classes	3 (18)	1 (11)	5 (45)	2 (18)	11 (23)
	Weight lifting	4 (24)	3 (33)	8 (73)	3 (27)	18 (38)
	Gardening	4 (24)	6 (68)	7 (64)	6 (55)	23 (48)
	Walked	13 (77)	5 (56)	10 (91)	9 (82)	37 (77)
	Water aerobics	6 (35)	1 (11)	2 (18)	3 (27)	12 (25)
**Frequency of physical activity, n (%)**						
	0 times per week	1 (6)	0 (0)	1 (10)	1 (9)	3 (6)
	Once per week	2 (12)	0 (0)	1 (10)	0 (0)	3 (6)
	2-3 times per week	6 (35)	4 (44)	3 (30)	4 (36)	17 (36)
	4-5 times per week	7 (41)	3 (33)	1 (10)	2 (18)	13 (28)
	More than 5 times a week	1 (6)	2 (22)	4 (40)	4 (36)	11 (23)
Average length of activity tracker use		0 months	Less than 3 months	10 months (before abandonment)	Over 12 months	8 months

**Table 3:** Technology ownership by focus group type.

Access to technology type	Nonusers, n (%)	Short-term users, n (%)	Former users, n (%)	Long-term users, n (%)	All users, n (%)
Access to a landline phone (N=46)	11 (65)	6 (89)	8 (72)	4 (36)	29 (63)
Access to a mobile phone (N=48)	15 (88)	8 (89)	11 (100)	10 (91)	44 (92)
Access to a desktop computer (N=48)	13 (77)	8 (89)	8 (72)	8 (72)	37 (77)
Access to internet-enabled laptop computer (N=47)	11 (65)	6 (78)	9 (81)	9 (81)	35 (75)
Access to tablet computer (N=48)	11 (65)	7 (78)	10 (91)	11 (100)	39 (81)
Access to an activity tracker (N=48)	0 (0)	9 (100)	8 (72)	11 (100)	28 (58)

As part of focus group discussion, participants talked about the meaning of health and the role of physical activity and activity trackers in such perceptions. There were little differences across groups as most participants associated being healthy with freedom and independence, ability to move, being in good mental health, having high-quality life, and holding positive attitudes (Multimedia Appendix 1).

Many participants occasionally referred to themselves as being *laggards* and *luddites*, that is, not being technologically savvy. Short-term users described themselves as *having health issues* and *being active*. Long-term users also mentioned having health issues but were more likely to describe themselves as being technologically savvy and early technology adopters. Talking about their favorite technology during the icebreaker, participants mostly referred to information and communication devices, such as desktop and laptop computers, mobile phones, and tablet computers. Technology access by focus group type is shown in Table 3. Notably, the majority of long-term tracker users did not have access to landline phones. Most participants associated activity trackers with pedometers and other health management technology.

### Ideal Tracker: Prettier, Bigger, and More Comfortable

RQs 1a and 2b asked about the perceptions of tracker features and functions. Nonusers before the activity tracker trial did not have any experience with the technology and many did not know what it was, so they could not clearly identify what features they favored. The only experience some nonusers had previously had with wearables was vicarious where they generated their knowledge through observing others. In their descriptions of an ideal tracker, nonusers expressed great interest in a possibility to *monitor diverse activities*, such as biking, water aerobics, golf, and others. Nonuser participants also suggested that an ideal activity tracker should count *calories burned*, monitor *heart rate*, *look good*, and be *user friendly*.

After trying activity trackers, nonusers found 4 features and functions to be useful: it could be used as a *watch* to show date and time, it had a *comfortable band*, it tracked *sleep*, and it was *waterproof*. Interestingly, the step count feature was not a favorite feature among the nonusers as most perceived step quantification to be inaccurate.

After the activity tracker trial, nonusers’ preferences for an ideal activity tracker notably changed. Although they continued to suggest that activity trackers should be aesthetically appealing and not *ugly*, they focused on activity tracker band comfort, which, according to many participants, Garmin VivoFit 2 lacked. Nonusers described the band as *plastic* (ie, cheap and of bad quality), *clunky*, *annoying*, *rigid*, and *uncomfortable*:

It’s very rigid. The design is poor. It collects water underneath. I end up having a really loose bracelet. Which could have some effect on accuracy. I don’t know. I found it totally uncomfortable. It’s really ugly.

Female participant, nonuser

Another characteristic of an ideal tracker that was mentioned by nonusers after the VivoFit 2 trial was related to design features for *better vision*. Those referred to multiple aspects of activity tracker display that made reading easier, from the size of symbols to the direction of symbol placement on the screen (horizontal vs vertical) and light.

Looking *nice*, *cool*, and *fashionable*, as well as looking like something else (eg, *watch*, *bracelet*, or *necklace*) and having a comfortable band were equally important for participants in all 4 types of focus groups. Short-term and former activity tracker users identified simple usage patterns by focusing on the importance of step count. They also wished that activity trackers were more *user friendly* for older adults when they discussed device maintenance:

Short-term users expressed an appreciation of heart rate monitoring feature and wished to have features for better vision and a *stopwatch* function on their ideal activity trackers. Former users liked the sleep-tracking feature and wanted an activity tracker that measures *blood pressure* and provides *accurate information*.

Long-term tracker users, who were in the maintenance stage, showed a much more diverse usage of activity trackers and had a more elaborate *wish list*. In addition to features popular in other focus groups, long-term users identified *motivational messages and reminders*, *pace tracking*, and *calories burned* as being important:

I use mine as a, as a watch, um, when I wear it. I use it for distance, I use it for time, and I use it for my pace and calories expended.

Female participant, long-term user

Time feature, in addition to physical activity tracking, was crucial to long-term users. They also wanted activity trackers to be waterproof, have longer *battery life*, track *physical progress* in real time, automatically count *calories consumed*, and measure heart rate.

### Maintenance: Trial, Opportunity, Togetherness, and Internal Motivation

RQs 2a and 2b asked about participants’ perceptions of activity tracker use benefits and motivational strategies that they used, would use, or had used to sustain tracker usage over time. The overarching idea expressed by nonusers before the activity tracker trial was that trackers *would not motivate* them to increase physical activity. Some nonusers suggested they would be interested in *increasing awareness* of their daily activity as it could encourage them to move more. *Goal setting*, or knowing exactly what outcome to strive for, could also strengthen the activity tracker use motivation. Several nonuser participants named *long-term benefits*, such as staying away from medications, feeling better, losing weight, and improving physical indicators of health (heart rate and blood pressure), as motivations that could drive tracker use.

After trying Garmin VivoFit 2, the *activity tracker does not motivate* theme became less pronounced. Participants agreed that tracker use motivated them to walk more, driven by quantifying activity (counting steps) and continuously making users “more conscious of extra walking” (female participant, nonuser):

I did find myself looking all the time, how many steps I had, and did try to. In a couple days, I had like 2,000 steps and felt guilty as all get out. I really enjoyed it. I didn't think I would, I thought maybe for a week or two, the novelty you know. In fact, I’m monitoring it as I came upstairs today, so I'm really enjoying it.

Male participant, nonuser

The overwhelming tactic that the nonusers used was to slightly *modify daily activities* to take more steps. Those included taking stairs instead of elevators, parking far away from destination buildings, or walking to an area instead of driving.

Short-term and former users talked about the same tactics that nonusers did, with an addition of 2 new *tricks*: using a *red-line* activity tracker alert of inactivity and shopping.

Short-term and former users were also motivated by the *presence of others*: either participants competed with their friends and family members in numbers of steps taken, were supported by them (eg, being given a tracker as a gift), or simply observed them using activity trackers:

I would say one of the things that I did, my kids had gotten some wearables, and so we had a little contest. That encouraged me, instead of having the red line or something like that. It was like, “Oh, so how many did you do today?” It was a non‑threatening way of getting everybody to see what they did, so, “You only did so many steps today? What's going on with you?”

Female participant, former user

The presence of others was the top motivator for long-term users. Although competition was an important motivator, the importance of “togetherness” and cooperation, that is, doing something enjoyable together to achieve individual fitness goals, was more prevalent in conversations with long-term users:

When we lived in Houston, and we both worked and carried pagers and cell phones or whatever, Friday night was date night and we started date night with a run together. And, cooled down, and then went out to dinner. So, it's part of our marriage in a way, and it was part of our dating relationship and that's when I think about, it's just like brushing your teeth every morning.

Female participant, long-term user

Achieving goals was very satisfactory to long-term users who derived gratifications from using activity trackers that confirmed completion of daily activity tasks and gave *rewards*:

It's just amazing when it tells me I've gotten my 10,000 steps in, I've gotten my ten stairs, I've burned my 2,000+ calories, whatever, and I get this little green flashy thing on my phone, it's stupid I get it, but I look for that at night. “Oh did I get my flashy thing?” Ya know? It's, it's motivating.

Female participant, long-term user

Long-term users were more likely than participants in other focus groups to see long-term benefits of regular physical activity and, consequently, activity tracker use, such as physical and emotional health (eg, stress reducer), reduced pain, independence and mobility, and long life:

When you start seeing your classmates in the obituaries, I think that...

Female participant, long-term user

It’s an eye-opener, isn’t it?

Female Participant, long-term user

Or you see them and you think, “Boy, do they look old!” [laughs] you know? “I don’t want to be that person...

Female participant, long-term user

Long-term users, compared with other participants, perceived external factors, such as change of weather, as *opportunities*. Seasons, especially summer, facilitated physical movement. One participant found an opportunity to be physically active in winter by shoveling snow (male participant, long-term user). Long-term users, however, expressed that external factors did not fully determine motivation. *Forcing* oneself, being consistently active day to day, allowing bad days but *reimbursing* for them later was indicative of *internal motivation* playing the most important role in maintaining physical activity. Technology came in second, only as a facilitator:

It’s something that clicks in your mind. You have to make that commitment, and once you do, the technology is very motivating. But, until you take the step of getting it and tuning into it, it isn’t going to work.

Female participant, long-term user

### What’s Stopping Them: Data Inaccuracy and Lack of Adequate Instructions

RQs 3a and 3b asked about perceived barriers to tracker use. Participants in the former and shot-term user focus groups appeared to like their routines and established exercise schedule, to which activity trackers did not add. To them, physical activity came before the use of the device. It was not that participants were not motivated to be active (they actually were), but activity tracker use did not seem to be the driver of that motivation:

[...] I've done 9,000 steps and it’s 8:30 at night and it’s my time to sit and quilt. I’m not going to do anymore. [laughs] I figured out I’ve done plenty.

Female participant, short-term user

Tracker nonusers mostly generated questions and speculations about how trackers worked: “Are they waterproof?”; “Does it monitor your sleep?”; “Do we have to keep it on our wrist?”; “Is it physically moving or is it your wrist that tracks us?”; “Does it tell time?”; and “Is it counting calories you’re using?” With the lack of knowledge came skepticism as nonusers questioned the ways in which activity trackers worked. Inaccuracy in counting steps raised most of the concerns. Participants explained that physical activities were very diverse and had different levels of intensity that no technology could grasp.

After the activity tracker trial was over, nonusers continued to ask questions about how the device worked. Their skepticism toward and disappointment with the technology increased because they found that activity trackers had given inaccurate information. It also had limitations in terms of features offered. Participants compared actual step recordings with their perceived activity and found obvious inconsistencies:

I thought they were highly inaccurate. I clocked 1,350 steps just driving out to eat in Rapids one day.

Female participant, nonuser

After the tracker trial, nonusers also suggested to improve activity tracker use *instructions* tailored to the population of older adults. They expressed the need to be better educated about various device features and functions, as well as synchronization with other devices, and suggested using step-by-step detailed instructions and in-group learning environment. In the first nonuser set of focus groups held in February, participants suggested that video instructions (similar to YouTube videos) would work well to guide and motivate older adults in tracker use. Nonuser participants in May groups, thus, were provided with such videos (see Methods). However, most May nonuser participants did not watch the videos, and those who watched them were not motivated more than they normally would be. Some participants experienced a classic case of the third-person effect [44]; they were sure that others “but not me” would be motivated by the videos.

Other reasons for nonadoption included high price, physical limitations that older adults face as well as inactive lifestyle, little interest and curiosity in trying activity trackers, band discomfort, and technology use difficulties. Technology-related barriers were discussed more extensively after the nonusers tried activity trackers, especially with regard to mobile phone ownership and synchronizing activity trackers with it.

Short-term and former users added that defects in activity trackers could or did make them stop using the activity tracker. One short-term user briefly discussed the issue of keeping personal data private when information from the activity tracker is synchronized with a mobile phone or computer and shared on social networks.

Former users indicated that long-term use of activity trackers was not necessary as one gets an impression of numbers related to certain activities. Many former users were found to maintain the same daily routines, so they quickly learned how many steps, calories, and miles daily activities were associated. After that, using an activity tracker was not a priority for them:

I know that I get about 13,000 steps. I don't use it every day. I started wearing it again when I said I would sign up for it just to make sure that I was still doing what I thought I was doing because I walk in the morning before I go to work, and then do my activities.

Female participant, former user

A few former users also indicated that having a short battery life and losing activity trackers were the reasons why they stopped using the device.

Conversations with nonusers and short-term and former users centered on barriers to adopt or continue using an activity tracker, but this theme was less pronounced in long-term users who predominantly discussed activity tracker features and motivations to use. No unique additional barriers were discovered in focus groups with long-term users.

## Discussion

### Principal Findings

#### From the Basics to Sophistication

The results indicated that focus group participants, who were aged 65 years and older, favored and expected their activity trackers to have a wider variety of features than what had been shown in previous research within this population [8,14]. Participants with different levels of activity tracker use experience liked and wished to use different activity tracker features. Long-term users who were in the maintenance stage of activity tracker use indicated a more diverse and sophisticated usage of activity trackers. This could be explained by the length and richness of experience with activity trackers as well as the higher level of technological savviness mentioned by the long-term users. Nonusers expressed disappointment with the technology as it did not meet their expectations regarding accurate step count and did not track various activities automatically. Health-related monitoring, such as weight watching, hear rate, and blood pressure were not the most popular features despite many participants reported having chronic conditions.

#### From Effort to Effortlessness

Nonusers and short-term users had to *trick* themselves into using activity trackers to countercondition inactivity by slight modifications in daily routines. Long-term users were more habitual, automatic, and effortless in their activity tracker use. Long-term users emphasized the importance of internal motivation (*Just do it*) where activity trackers were serving as secondary facilitators and expressed the enthusiasm about modifying the environment to *keep going*.

#### Maintenance Through Internal Motivation

It is recommended to focus on the importance of intrinsic over extrinsic motivation for tracker use maintenance in future studies. The distinction between the 2 types of motivation is made within the framework of self-determination theory (SDT). SDT studies have consistently demonstrated that intrinsic motivations are predictors of long-term physical activity adherence and weight loss [45,46]. SDT posits that motivation is driven by individuals striving to satisfy 3 essential needs: autonomy (independence in the world of external constraints), competence (self-efficacy), and social relatedness [47-49]. Intrinsic motivation refers to engaging in an activity out of one’s authentic interest in it, which brings inherent satisfaction and feelings of enjoyment, accomplishment, and excitement [47,50]. For example, the long-term users in this study not only emphasized the *Just Do It* aspect of sustained activity tracker use but also referred to positive emotional rewards they would get after completing their goals. Extrinsic motivation refers to engaging in activities that provide rewards from the outside. Extrinsic motivation with a greater degree of autonomy (eg, increased socialization via the activity tracker network) leads to a higher likelihood of behavior maintenance than external motivation with low autonomy levels (eg, activity tracker use because of a doctor’s prescription) that is only effective in the short run [47,51-55]. The results of this study suggest that long-term use of activity trackers depends mostly on intrinsic motivation and extrinsic motivation at greater level of autonomy, suggesting that this behavior can be successfully maintained over time. Satisfaction of the social relatedness need through tracker use is another powerful driver of maintenance.

#### Observe-Compete-Collaborate

Social support was not a pronounced motivation for nonusers who often observed others using activity trackers, and it was only secondary for short-term and former users who emphasized the importance of competition with others. Long-term users indicated social support to be the main motivational factor, with the focus on building relationships around daily activity routines. Long-term users were better prepared to modify the social environment around them to maintain an active lifestyle, receive positive feedback, and seek accountability from others.

#### Awareness Before Health Benefits

Short-term, long-term, and former participants exhibited high levels of physical activity, which could have contributed to curiosity about activity tracking but not to the understanding of its health management value. Nonusers were unaware of tracker potential in helping to meet physical activity goals. Rising consciousness occurred among some tracker nonusers as they realized that activity tracker use could benefit them in terms of feeling better, losing weight, and lowering blood pressure. Nonusers reported lower physical activity frequency and said activity tracker use helped them increase physical activity levels. This result suggests that tracker use could be more useful for those older adults who are not physically active and seek to change that. Our short-term and former users who showed high levels of physical activity indicated greater skepticism and disappointment with the technology. Activity trackers provided them with information about physical activity, but it did not motivate to make already active users be more active. It could be that because activity trackers did not drastically change physical activity levels in the group of former users, they failed to maintain its use.

#### Achieving a Pros-and-Cons Balance

Long-term users were more engaged in the discussion of activity tracker features and motivations, whereas participants in other groups focused on problems with the activity tracker related to the lack of activity tracker knowledge and tracker use skills as well as inaccuracy and defects in activity tracker measures. Long-term users also saw many more benefits of activity tracker use than their counterparts from other focus groups. This result is in line with the proposition of the TTM that the maintenance stage of behavior change is associated with seeing a greater number of benefits than costs [18]. Former users not only reported the same high level of physical activity as short-term and long-term users but also exhibited a greater diversity of physical activity, which indicated that the activity tracker was not the primary motivator.

### Comparison With Previous Work

This study contributes to the existing knowledge about wearable technology use in 4 meaningful ways. First, we analyzed the experiences of older adults at different levels of tracker use with a special focus on the maintenance stage of behavior change and relapse (ie, discontinuing tracker use) [16]. Previous studies have explored activity tracker use mostly at the initial stages of use [2,8,10,56] and have not systematically evaluated the spectrum of features used, liked, and wanted by adults aged 65 years and older. In addition, little research has been previously done on tracker use relapse in older adults, and our study filled that gap.

Second, we outlined several new activity tracker features and characteristics that adults in the *65+* age group would want to benefit from on a long-term basis. One of the significant findings of this study was that our participants valued tracker device comfort and aesthetics more than the basic features of step and calorie count, sleep and heart rate tracking, and water resistance that were found important for older adults in previous research [8,11]. The suggestion of making the activity tracker accommodate the older target groups in terms of features for better vision came afterward. A possible explanation of these results could be that older adults felt underappreciated by the current consumer technology developers that often target younger populations. Thus, they required devices to meet basic consumer needs in comfort and pleasant appearance to break the association with *bulky* and *ugly* devices *for older adults*. Another possibility is that our sample is skewed to be more highly educated than the average older adults; thus, their expectations may vary in terms of activity tracker characteristics. Further research with more diverse samples of older adults might yield different findings.

Third, this study indicated that health benefits provided to adults who are aged 65 years and older by activity tracker use were not greatly important to them. Participants in all focus groups put awareness of physical activity and curiosity as the primary reasons to start and continue activity tracker use. In general, the motivators to continuously use the tracker mirrored those mentioned in previous literature: physical activity awareness, goal setting, positive reinforcement, and social connection [1-4,8,10,29-34,57,58]. Participants in our study, like participants in other studies [59], saw the benefits of physical activity awareness via self-monitoring and goal setting. Our findings regarding social support indicated that social support is not only one of the most important and consistent predictors of physical activity adherence [60-63] but also a crucial factor to maintain tracker use. Furthermore, we found that nonusers expected activity trackers to track diverse activities and were disappointed that the technology did not do it. This is echoed by the findings of another study advocating for a more tailored approach to tracking activities salient to older adults [64].

Finally, this study added to the list of the known barriers to activity tracker technology adoption and continued use and explained why relapse in activity tracker use happens. In addition to technology defects, lack of technology use skills, physical and psychological limitations, and financial restrictions [35,65], our participants added distrust in the activity tracker’s capability of accurate data tracking. The questions that participants posed about activity tracker functions, speculations about how this technology works, and skepticism about it indicated a need for future studies of older adults’ technology literacy [66]. Former users were motivated by knowing the numbers behind their daily physical routines, but they were not eager to use activity trackers to modify or increase these daily physical activities.

### Study Limitations and Suggestions for Future Research

Though our study was extensive in the type of activity tracker users included, the sample was more educated, slightly younger, and less ethnically and socioeconomically diverse than older adults in general. For example, 23% of older adults in the *65+* age group in the United States were members of racial or ethnic minority populations in 2016 [67], which is higher than the percentage of our sample participants who were not white (less than 15%). In terms of educational attainment, approximately 86% of older adults completed high school and about 30% completed a bachelor’s degree or higher in 2017 [67]. Our sample was highly educated, with over 40% of participants holding a graduate degree. Furthermore, 44 of the 48 participants (92%) had access to a mobile phone, which is higher than the national average of 78% [42]; 39 out of 48 (81%) had access to a tablet computer, which is also higher than the national rate for older adults (32%) [42]; and 36 out of 47 (75%) reported having access to an internet-enabled laptop computer, which is also higher than the national average of 55% [42]. Higher levels of education, income, technology access, younger age, and greater homogeneity in terms of race or ethnicity could influence the level of physical activity in older adults and, as a result, emphasize certain perceptions of activity trackers detected in this study. For example, only 6% of our participants did not engage in physical activity and another 6% engaged in it only once a week. This is much lower than the national average where 84% of adults aged 65 years and older live sedentary lifestyles [24-26]. Perhaps demographic and physical activity characteristics of our sample led to skeptical perceptions of activity trackers, emphasis on internal motivation to maintain activity tracker use and physical activity, as well as viewing the activity tracker as the tool to increase the awareness of physical activity rather than physical activity itself. That is why, possibly, activity tracker use was found to be most beneficial to nonusers who were less active and had lower technology access. Future studies should focus on sedentary older populations to explore activity tracker use maintenance and relapse. Studies with more diverse samples of older adults might reveal other facilitators and barriers to using activity trackers. In addition, the study solely focused on adults who are aged 65 years and older and did not collect empirical data from younger activity tracker users. This shortcoming should be addressed in future studies.

We had difficulty recruiting short-term tracker users. This leads us to question activity tracker uptake among older adults, at least in the Midwest state where this study took place. To our surprise, it was easier to find participants who had been using activity trackers for over 6 months than those who had just started using this technology. In addition, we used only 1 type of activity tracker with our nonuser participants. The limitations of Garmin Vivofit 2 could have had an effect on nonusers’ evaluations of activity trackers in general.

Finally, including participants with specific types of chronic diseases could yield specific activity tracker use facilitators and barriers for these groups that might influence future studies. We were not able to focus on specific chronic disease groups in this study. Long-term randomized controlled trials that incorporate multiple-level interventions are needed to enhance physical activity among older adults.

### Practical Implications

The findings of this study have several practical implications for activity tracker design and health interventions involving activity trackers. Many commercial trackers do not come with a detailed manual other than simple book leaflets and a URL for the users to refer to. It is assumed that the use is intuitive, or users can use the internet to find additional information. As one of the primary barriers to the device adoption among older adults was lack of knowledge, activity tracker manufacturers or researchers attempting to use the technology as health promotion devices may consider developing detailed manuals with screen captures and visual illustrations of features, buttons, and navigation in the app, especially if the target users are older adults who are used to having a hard-copy manual. Step-by-step instructions of use may be a good way to educate older adult users who are not technologically savvy [68]. If the promotional materials of the activity tracker manufacturers highlight the ease of use, older adults may be more likely to take an action to adopt the activity tracker.

Skepticism regarding the accuracy of activity trackers and their ability to capture various physical activities was another major barrier to adoption and activity tracker use maintenance. Activity tracker designers and manufacturers could include in their promotional materials explanations of how the activity tracker works and acknowledge that activity trackers cannot capture all activities including bicycling and swimming because of the nature of the accelerometers used in activity trackers for tracking activity. However, users should be able to manually enter the data of these activities not captured by trackers and still get an estimate of equivalent “step count” based on type, intensity, and duration.

Physical appearance and comfort were cited by many users, both long-term and short-term. Therefore, making the band of the activity tracker to be similar to a bracelet or another piece of jewelry that can match outfits or express identity seems to be a good strategy. This may be especially useful when individuals are already at the maintenance stage of behavior change to keep them engaged.

Many former users complained about the battery life of activity trackers, and it was identified as one of the reasons for abandonment. Battery capacity is an issue when the activity tracker is designed to be small. However, as we know that older adults prefer to have a bigger screen, we suggest that when activity tracker designers are faced with the dilemma of a small-sized activity tracker and larger battery capacity, they should put priority on battery life when creating technology for older populations to ensure sustained use.

Social support was considered to be a major facilitating factor among long-term users to use activity trackers to keep physically active. Although friendly competition was mentioned by some long-term participants, the majority of them relied on not competition but cooperation or collaboration—working together to be physically active. This indicates that activity tracker designers may add features to activity trackers and their associated apps to facilitate social support and the concept of working out or walking together with family and friends.

### Conclusions

Our research suggests that there is no *magic bullet* approach to ensuring that older adults will use activity trackers on a long-term basis. Focusing on individual-, interpersonal-, and community-level factors that predict the maintenance of activity tracker use behavior will likely be needed to help older adults effectively use activity trackers to become and stay more physically active. In addition, activity tracker developers and manufacturers should consider the design aspects that may be most relevant for older adults, given the rapidly increasing size of this demographic group around the world.

## Appendix

Multimedia Appendix 1 Number of sources (focus groups) and references (mentions) for codes and code categories.

## Abbreviations

RQ: research question
SDT: self-determination theory
TTM: transtheoretical model

## References

[1] Cadmus-Bertram LA, Marcus BH, Patterson RE, Parker BA, Morey BL (2015). Randomized trial of a Fitbit-based physical activity intervention for women. Am J Prev Med.

[2] O'Brien T, Troutman-Jordan M, Hathaway D, Armstrong S, Moore M (2015). Acceptability of wristband activity trackers among community dwelling older adults. Geriatr Nurs.

[3] Mercer K, Li M, Giangregorio L, Burns C, Grindrod K (2016). Behavior change techniques present in wearable activity trackers: a critical analysis. JMIR Mhealth Uhealth.

[4] Lyons EJ, Lewis ZH, Mayrsohn BG, Rowland JL (2014). Behavior change techniques implemented in electronic lifestyle activity monitors: a systematic content analysis. J Med Internet Res.

[5] (2008). US Department of Health and Human Services.

[6] Levine DM, Lipsitz SR, Linder JA (2016). Trends in seniors' use of digital health technology in the United States, 2011-2014. J Am Med Assoc.

[7] Ledger D, McCaffrey D (2014). Endeavour Partners.

[8] Mercer K, Giangregorio L, Schneider E, Chilana P, Li M, Grindrod K (2016). Acceptance of commercially available wearable activity trackers among adults aged over 50 and with chronic illness: a mixed-methods evaluation. JMIR Mhealth Uhealth.

[9] Puri A, Kim B, Nguyen O, Stolee P, Tung J, Lee J (2017). User acceptance of wrist-worn activity trackers among community-dwelling older adults: mixed method study. JMIR Mhealth Uhealth.

[10] Bravata DM, Smith-Spangler C, Sundaram V, Gienger AL, Lin N, Lewis R, Stave CD, Olkin I, Sirard J (2007). Using pedometers to increase physical activity and improve health: a systematic review. J Am Med Assoc.

[11] Li L, Peng W, Kamp K, Bowen M, Cotten S, Rikard R, Kononova A (2017). Poster: Understanding long-term adoption of wearable activity trackers among older adults.

[12] Mercer K, Li M, Grindrod KA (2015). Do wearable activity trackers have a place in pharmacies?. Can Pharm J (Ott).

[13] Karapanos E, Gouveia R, Hassenzahl M, Forlizzi J (2016). Wellbeing in the making: peoples' experiences with wearable activity trackers. Psychol Well Being.

[14] (2015). American Association of Retired Persons (AARP) CATALYST.

[15] Prochaska JO, DiClemente CC (1983). Stages and processes of self-change of smoking: toward an integrative model of change. J Consult Clin Psychol.

[16] Prochaska JO, Reddings CA, Evers KE, Glanz K, Rimer BK, Viswanath K (2008). The transtheoretical model and stages of change. Health Behavior and Health Education: Theory, Research, and Practice.

[17] Prochaska JO, Velicer WF (1997). The transtheoretical model of health behavior change. Am J Health Promot.

[18] Prochaska J (2008). Decision making in the transtheoretical model of behavior change. Med Decis Making.

[19] Asimakopoulos S, Asimakopoulos G, Spillers F (2017). Motivation and user engagement in fitness tracking: heuristics for mobile healthcare wearables. Informatics.

[20] Manini TM, Pahor M (2009). Physical activity and maintaining physical function in older adults. Br J Sports Med.

[21] (1999). Centers of Disease Control and Prevention.

[22] Nelson ME, Rejeski WJ, Blair SN, Duncan PW, Judge JO, King AC, Macera CA, Castaneda-Sceppa C, American College of Sports Medicine, American Heart Association (2007). Physical activity and public health in older adults: recommendation from the American College of Sports Medicine and the American Heart Association. Circulation.

[23] Haskell WL, Lee IM, Pate RR, Powell KE, Blair SN, Franklin BA, Macera HG, Heath GW, Thompson PD, Bauman A (2007). Physical activity and public health: updated recommendation for adults from the American College of Sports Medicine and the American Heart Association. Med Sci Sports Exerc.

[24] Craig CL, Russell SJ, Cameron C, Bauman A (2004). Twenty-year trends in physical activity among Canadian adults. Can J Public Health.

[25] Caspersen CJ, Pereira MA, Curran KM (2000). Changes in physical activity patterns in the United States, by sex and cross-sectional age. Med Sci Sports Exerc.

[26] Bennett J, Winters-Stone K, Nail L, Scherer J (2006). Definitions of sedentary in physical-activity-intervention trials: a summary of the literature. J Aging Phys Act.

[27] Center for Disease Control and Prevention.

[28] Elsawy B, Higgins KE (2010). Physical activity guidelines for older adults. Am Fam Physician.

[29] Michie S, Abraham C, Whittington C, McAteer J, Gupta S (2009). Effective techniques in healthy eating and physical activity interventions: a meta-regression. Health Psychol.

[30] Newall NE, Menec VH (2017). Loneliness and social isolation of older adults: why it is important to examine these social aspects together. J Soc Pers Relationships.

[31] Fan C, Forlizzi J, Dey A (2012). Considerations for technology that support physical activity by older adults. Proceedings of the 14th international ACM SIGACCESS conference on Computers and accessibility.

[32] Randriambelonoro M, Chen Y, Pu P (2017). Can fitness trackers help diabetic and obese users make and sustain lifestyle changes?. Computer.

[33] Sookhai L, Coppola JF, Gaur C (2015). Intergenerational activity tracker program: impact with health related outcomes on older adults. https://www.researchgate.net/publication/315956465_Can_Fitness_Trackers_Help_Diabetic_and_Obese_Users_Make_and_Sustain_Lifestyle_Changes.

[34] Wang JB, Cadmus-Bertram LA, Natarajan L, White MM, Madanat H, Nichols JF, Ayala GX, Pierce JP (2015). Wearable sensor/device (Fitbit One) and SMS text-messaging prompts to increase physical activity in overweight and obese adults: a randomized controlled trial. Telemed J E Health.

[35] Ehn M, Eriksson LC, Åkerberg N, Johansson AC (2018). Activity monitors as support for older persons' physical activity in daily life: qualitative study of the users' experiences. JMIR Mhealth Uhealth.

[36] Compernolle S, Vandelanotte C, Cardon G, De Bourdeaudhuij I, De Cocker K (2015). Effectiveness of a web-based, computer-tailored, pedometer-based physical activity intervention for adults: a cluster randomized controlled trial. J Med Internet Res.

[37] Devereux-Fitzgerald A, Powell R, Dewhurst A, French DP (2016). The acceptability of physical activity interventions to older adults: a systematic review and meta-synthesis. Soc Sci Med.

[38] Hermsen S, Moons J, Kerkhof P, Wiekens C, De Groot M (2017). Determinants for sustained use of an activity tracker: observational study. JMIR Mhealth Uhealth.

[39] Meyer J, Schnauber J, Heuten W, Wienbergen H, Hambrecht R, Appelrather HJ, Boll S (2016). Exploring longitudinal use of activity trackers.

[40] Valerio MA, Rodriguez N, Winkler P, Lopez J, Dennison M, Liang Y, Turner BJ (2016). Comparing two sampling methods to engage hard-to-reach communities in research priority setting. BMC Med Res Methodol.

[41] Bonevski B, Randell M, Paul C, Chapman K, Twyman L, Bryant J, Brozek I, Hughes C (2014). Reaching the hard-to-reach: a systematic review of strategies for improving health and medical research with socially disadvantaged groups. BMC Med Res Methodol.

[42] Guetterman TC (2015). Descriptions of sampling practices within five approaches to qualitative research in education and the health sciences. Forum Qual Soc Res.

[43] Feng GC (2014). Intercoder reliability indices: disuse, misuse, and abuse. Qual Quant Int J Methodol.

[44] Davison WP (1983). The third-person effect in communication. Public Opin Q.

[45] Mata J, Silva MN, Vieira PM, Carraça EV, Andrade AM, Coutinho SR, Sardinha LB, Teixeira PJ (2009). Motivational "spill-over" during weight control: increased self-determination and exercise intrinsic motivation predict eating self-regulation. Health Psychol.

[46] Teixeira PJ, Carraça EV, Markland D, Silva MN, Ryan RM (2012). Exercise, physical activity, and self-determination theory: a systematic review. Int J Behav Nutr Phys Act.

[47] Ryan RM, Deci EL (2000). Self-determination theory and the facilitation of intrinsic motivation, social development, and well-being. Am Psychol.

[48] Ryan RM, Deci EL (2000). The darker and brighter sides of human existence: basic psychological needs as a unifying concept. Psychol Inq.

[49] Silva de Lima AL, Hahn T, de Vries NM, Cohen E, Bataille L, Little MA, Baldus H, Bloem BR, Faber MJ (2016). Large-scale wearable sensor deployment in Parkinson's patients: the Parkinson@home study protocol. JMIR Res Protoc.

[50] Deci E (1975). Intrinsic Motivation.

[51] Deci E, Ryan R, Richard M (1985). Intrinsic Motivation And Self-Determination In Human Behavior.

[52] Ryan RM, Plant RW, O'Malley S (1995). Initial motivations for alcohol treatment: relations with patient characteristics, treatment involvement, and dropout. Addict Behav.

[53] Williams GC, Freedman ZR, Deci EL (1998). Supporting autonomy to motivate patients with diabetes for glucose control. Diabetes Care.

[54] Williams GC, Grow VM, Freedman ZR, Ryan RM, Deci EL (1996). Motivational predictors of weight loss and weight-loss maintenance. J Pers Soc Psychol.

[55] Williams GC, Rodin GC, Ryan RM, Grolnick WS, Deci EL (1998). Autonomous regulation and long-term medication adherence in adult outpatients. Health Psychol.

[56] McMahon SK, Lewis B, Oakes M, Guan W, Wyman JF, Rothman AJ (2016). Older adults' experiences using a commercially available monitor to self-track their physical activity. JMIR Mhealth Uhealth.

[57] Olanrewaju O, Kelly S, Cowan A, Brayne C, Lafortune L (2016). Physical activity in community dwelling older people: a systematic review of reviews of interventions and context. PLoS One.

[58] van Stralen MM, De Vries H, Mudde AN, Bolman C, Lechner L (2009). Determinants of initiation and maintenance of physical activity among older adults: a literature review. Health Psychol Rev.

[59] Mansi S, Milosavljevic S, Tumilty S, Hendrick P, Higgs C, Baxter DG (2015). Investigating the effect of a 3-month workplace-based pedometer-driven walking programme on health-related quality of life in meat processing workers: a feasibility study within a randomized controlled trial. BMC Public Health.

[60] Bauman AE, Reis RS, Sallis JF, Wells JC, Loos RJ, Martin BW, Lancet Physical Activity Series Working Group (2012). Correlates of physical activity: why are some people physically active and others not?. Lancet.

[61] Eyler AA, Brownson RC, Donatelle RJ, King AC, Brown D, Sallis JF (1999). Physical activity social support and middle- and older-aged minority women: results from a US survey. Soc Sci Med.

[62] Maher CA, Lewis LK, Ferrar K, Marshall S, De Bourdeaudhuij I, Vandelanotte C (2014). Are health behavior change interventions that use online social networks effective? A systematic review. J Med Internet Res.

[63] Rovniak LS, Kong L, Hovell MF, Ding D, Sallis JF, Ray CA, Kraschnewski JL, Matthews SA, Kiser E, Chinchilli VM, George DR, Sciamanna CN (2016). Engineering online and in-person social networks for physical activity: a randomized trial. Ann Behav Med.

[64] Fausset CB, Mitzner TL, Price CE, Jones BD, Fain BW, Rogers WA (2013). Older adults’ use of and attitudes toward activity monitoring technologies. Proc Hum Factors Ergon Soc Annu Meet.

[65] Floegel TA, Giacobbi Jr PR, Dzierzewski JM, Aiken-Morgan AT, Roberts B, McCrae CS, Marsiske M, Buman MP (2015). Intervention markers of physical activity maintenance in older adults. Am J Health Behav.

[66] Sullivan AN, Lachman ME (2016). Behavior change with fitness technology in sedentary adults: a review of the evidence for increasing physical activity. Front Public Health.

[67] US Department of Health and Human Services, Administration for Community Living Administration for Community Living.

[68] Cotten SR, Yost EA, Berkowsky RW, Winstead V, Anderson WA (2016). Designing Technology Training for Older Adults in Continuing Care Retirement Communities.

