# Supplementary material for: The Use of Wearable Activity Trackers Among Older Adults: Focus Group Study of Tracker Perceptions, Motivators, and Barriers in the Maintenance Stage of Behavior Change
Source: JMIR Mhealth Uhealth. 2019 Apr 5;7(4):e9832. doi: 10.2196/mhealth.9832 (PMC6473213; doi:10.2196/mhealth.9832)
Supplement: Multimedia Appendix 1 [file mhealth_v7i4e9832_app1.docx]

Multimedia Appendix. Number of sources (focus groups) and references (mentions) for codes and code categories

| Themes | | | | | Nonusers before tracker trial | Nonusers after tracker trial | Short-term users | Former users | Long-term users | Total |
| --- | --- | --- | --- | --- | --- | --- | --- | --- | --- | --- |
| Ideal tracker: Prettier, bigger, and more comfortable | | | | | | | | | | |
|  | Wish list of activity tracker features | | | | 2/55 | 2/61 | 2/45 | 2/62 | 2/75 | 10/298 |
|  |  | Comfortable band | | | 1/4 | 1/13 | 2/5 | 2/8 | 2/8 | 8/38 |
|  |  | Aesthetics | | | 2/5 | 2/7 | 2/6 | 1/3 | 2/10 | 9/31 |
|  |  | Better vision features | | | 1/4 | 2/13 | 2/5 | 1/1 | 2/6 | 8/29 |
|  |  | Ease of use | | | 2/3 | — | ¼ | 1/8 | 1/5 | 5/20 |
|  |  | Tracking unique activities | | | 2/10 | 1/2 | 1/3 | 1/4 | 1/1 | 6/20 |
|  |  | Compatibility with other devices | | | 1/1 | 1/3 | 1/3 | 1/4 | 2/6 | 6/17 |
|  |  | Waterproof | | | 1/1 | — | 1/3 | 1/4 | 2/4 | 5/12 |
|  |  | Calorie count | | | 2/6 | — | 1/1 | 1/2 | 2/2 | 6/11 |
|  |  | Motivational messages, alerts | | | - | 1/3 | 1/2 | 1/3 | 2/3 | 5/11 |
|  |  | Device size | | | — | 1/1 | 2/2 | 1/4 | 1/3 | 5/10 |
|  |  | Heart rate and pulse | | | 1/6 | — | — | 1/1 | 2/2 | 4/9 |
|  |  | Battery, charging | | | — | 1/1 | 1/3 | 1/2 | 2/3 | 5/9 |
|  |  | Activity tracker as a watch | | | 1/2 | — | — | — | 2/6 | 3/8 |
|  |  | Accurate, reliable | | | 1/1 | 1/1 | — | 1/4 | 1/1 | 4/7 |
|  |  | Sleep tracking | | | 1/3 | — | 1/1 | — | 1/2 | 3/6 |
|  |  | Multifunctionality | | | 1/2 | 1/2 | 1/2 | — | — | 4/6 |
|  |  | Customization | | | 1/1 | — | — | 1/3 | 2/2 | 4/6 |
|  |  | Tracking physical progress | | | — | — | — | 1/1 | 2/3 | 3/4 |
|  |  | Voice recognition | | | 1/1 | — | — | — | 1/2 | 2/3 |
|  | Activity tracker features used and liked | | | | 1/3 | 2/27 | 2/23 | 2/22 | 2/73 | 9/148 |
|  |  | | | Steps, walking | 1/1 | 1/1 | 1/4 | 2/6 | 2/14 | 7/26 |
|  |  | | | Sleep tracking | — | 2/3 | 2/2 | 2/6 | 2/7 | 8/18 |
|  |  | | | Heart rate and pulse | — | 1/2 | 1/6 | — | 2/9 | 4/17 |
|  |  | | | Activity tracker as a watch | — | 2/5 | 1/1 | — | 2/9 | 5/15 |
|  |  | | | Band | 1/2 | 2/3 | 1/1 | 1/2 | 2/4 | 7/12 |
|  |  | | | Waterproof | — | 2/3 | 1/2 | — | 2/5 | 5/10 |
|  |  | | | Compatibility with other devices | — | 1/2 | 2/2 | 1/1 | 2/4 | 6/9 |
|  |  | | | Motivational messages, alerts | — | — | 1/2 | — | 2/7 | 3/9 |
|  |  | | | Tracking distance over time | — | 1/1 | — | — | 2/5 | 3/6 |
|  |  | | | Battery and charging | — | 1/1 | — | 1/2 | 1/2 | 3/5 |
|  |  | | | Calories burned | — | — | — | — | 2/2 | 2/2 |
|  | Activity tracker features that are not used | | | | — | 2/4 | 1/1 | 2/3 | 2/22 | 7/30 |
|  |  | | | Sleep tracking |  | 1/1 |  | — | 2/7 | 3/8 |
|  |  | | | Calories consumed |  | 1/1 | — | 1/1 | 2/4 | 4/6 |
|  |  | | | Stopwatch |  | 2/2 | — |  | 1/2 | 3/4 |
| Maintaining activity tracker use: Trial, togetherness, opportunity, and internal motivation | | | | | | | | | | |
|  | Reasons to launch activity tracker use | | | | 2/17 | 2/4 | 2/10 | 2/10 | 2/20 | 10/61 |
|  |  | | | Awareness of activity | 1/4 | 2/3 | 2/5 | 1/3 | 2/4 | 8/19 |
|  |  | | | Curiosity | 2/6 | 1/1 | 2/3 | — | — | 5/10 |
|  |  | | | Become more active | 1/1 | — | 1/1 | 1/2 | 2/2 | 5/6 |
|  |  | | | Improve health, manage illness | — | — | — | 2/2 | 2/5 | 4/7 |
|  |  | | | Seeing success of others | — | — | — | 2/2 | 2/3 | 4/5 |
|  |  | | | Lose weight | ½ | — | — | — | 1/1 | 2/3 |
|  | Motivation | | | | 2/30 | 2/45 | 2/44 | 2/43 | 2/141 | 10/303 |
|  |  | | | Awareness of activity | 1/5 | 2/16 | 2/6 | 2/8 | 2/25 | 9/60 |
|  |  | | | Long-term health benefits | 1/4 | 1/9 | 2/3 | 1/2 | 2/25 | 7/43 |
|  |  | | | Activity tracker use as a competition, game | 1/2 | 1/2 | 2/8 | 1/9 | 2/21 | 7/42 |
|  |  | | | Social support | 1/2 | 1/3 | 2/8 | 2/3 | 2/26 | 8/42 |
|  |  | | | Activity tracker does not motivate | 2/7 | 1/4 | 2/9 | 2/15 | 2/2 | 9/37 |
|  |  | | | Goal setting | 1/3 | 1/6 | 2/5 | — | 2/20 | 6/34 |
|  |  | | | Seasonal and weather changes | — | 1/1 | — | 1/1 | 2/8 | 4/10 |
|  |  | | | Internal motivation | 1/2 | — | — | 1/1 | 2/4 | 4/7 |
|  |  | | | Health condition, illness | 1/1 | 1/1 | 1/2 | — | 1/2 | 4/6 |
|  |  | | | Life changes | — | — | — | — | 2/4 | 2/4 |
|  | Forming an activity tracker use habit | | | | 2/4 | 1/6 | 2/6 | 2/9 | 2/38 | 9/63 |
|  |  | | | Creating an opportunity | 1/2 | 1/3 | — | 2/5 | 1/8 | 5/18 |
|  |  | | | Creating and using prompts | — | — | 2/2 | 1/2 | 1/5 | 4/9 |
|  |  | | | It’s hard to start | — | — | — | — | 2/6 | 2/6 |
| What’s stopping them: data inaccuracy as a demotivator | | | | | | | | | | |
|  | Barriers | | | | 2/55 | 2/59 | 2/59 | 2/54 | 2/50 | 10/277 |
|  |  | | | Accuracy | 2/32 | 2/32 | 2/30 | 1/25 | 2/11 | 9/130 |
|  |  | | | Technology hard to use | 2/3 | 2/11 | 2/11 | 2/6 | 2/3 | 10/34 |
|  |  | | | Instructions | 1/1 | 2/9 | 1/1 | 1/2 | 2/4 | 7/17 |
|  |  | | | Physical limitations | ½ | 1/4 | 2/5 | 1/2 | 2/4 | 7/17 |
|  |  | | | Poor battery quality | — | — | 1/1 | 2/9 | 2/6 | 5/16 |
|  |  | | | Price | 1/6 | 1/4 | 1/3 | 1/2 | — | 4/15 |
|  |  | | | Emotional | 1/5 | 1/3 | — | 1/1 | 2/4 | 5/13 |
|  |  | | | No interest | 1/5 | 1/3 | — | — | — | 2/8 |
|  |  | | | Physical activity is not a priority | 1/1 | 1/1 | 1/2 | 1/2 | 1/1 | 5/7 |
|  |  | | | Environmental | — | 1/2 | 1/1 | 1/2 | 1/1 | 4/6 |
|  |  | | | Lack of privacy | — | — | 1/4 | — | — | 1/4 |
|  | Reasons to stop activity tracker use | | | | 2/4 | 2/8 | 2/7 | 2/37 | 1/2 | 9/58 |
|  |  | | | Activity tracker defect | 1/1 | — | 2/3 | 2/16 | — | 5/15 |
|  |  | | | Awareness of activity reached | 1/2 | — | — | 2/6 | — | 3/8 |
|  |  | | | Not compatible with other devices | — | — | 1/1 | 1/2 | — | 4/8 |
|  |  | | | Lost activity tracker | — | 2/5 | — | 2/3 | — | 2/3 |
|  |  | | | Inaccurate data | — | 1/1 | 1/1 | 1/2 | — | 3/4 |
|  |  | | | Uncomfortable band | — | 1/2 | 1/1 | 2/2 | — | 4/5 |
|  |  | | | Activity tracker does not meet expectations | — | — | — | 1/1 | 1/1 | 2/2 |
| Sample characteristics | | | | | | | | | | |
|  | Meaning of health^b^ | | | | 2/33 | — | 2/17 | 2/33 | 2/34 | 8/117 |
|  |  | | | Freedom and independence | 2/7 |  | 2/5 | 2/8 | 2/9 | 8/29 |
|  |  | | | Moving | 2/9 | — | 2/4 | 2/6 | 2/7 | 8/26 |
|  |  | | | Mental health | 2/6 | — | 1/1 | 1/1 | 1/5 | 5/13 |
|  |  | | | Life quality | 2/4 | — | 2/3 | 2/4 | 2/2 | 8/13 |
|  |  | | | Social engagement | 1/1 | — | 2/2 | 2/4 | 1/1 | 6/8 |
|  |  | | | Being happy | 1/1 | — | 1/1 | 2/3 | 1/2 | 5/7 |
|  |  | | | Eating well | — | — | — | 2/2 | 2/5 | 4/7 |
|  |  | | | Not taking medicines | 1/1 | — | — | 2/3 | — | 3/4 |
|  | Participant self-descriptions^b^ | | | | 2/15 | 2/4 | 2/13 | 2/21 | 2/22 | 10/75 |
|  |  | | | Not tech savvy, laggard, luddite | 2/3 |  | 1/1 | 2/8 | 2/6 | 7/18 |
|  |  | | | Having had health issues | 1/1 | 1/1 | 2/5 | 1/5 | 2/2 | 7/14 |
|  |  | | | Early adopter, tech savvy | 1/1 | — | 1/1 | — | 2/6 | 4/8 |
|  |  | | | Having been active | 1/1 | — | 2/4 | 1/2 | 1/1 | 5/8 |
|  | Favorite technology* | | | | 2/18 | — | 1/4 | 2/16 | 2/24 | 7/62 |
|  | Computer (laptop, desktop) | | | | 2/3 | — | 1/1 | 2/7 | 2/9 | 7/20 |
|  | Phone | | | | 2/2 | — | 1/3 | 2/3 | 2/9 | 7/17 |
|  | Tablet computer | | | | 2/4 | — | — | 2/3 | 2/4 | 6/11 |
|  | E-reader | | | | — | — | — | 1/2 | — | 1/2 |
|  | Activity tracker associations^b^ | | | | 2/8 | 1/4 | 1/2 | 2/21 | 2/11 | 8/46 |
|  |  | | Activity tracker as a pedometer | | 1/1 | — | 1/1 | 2/9 | 2/2 | 6/13 |
|  |  | | Other technology as activity tracker (apps) | | — | — | — | 2/9 | 2/6 | 5/15 |
|  |  | | Wristband | | 1/1 | — | — | 1/2 | — | 2/3 |
|  |  | | Health | | 1/1 | 1/1 | — | — | — | 2/2 |

*Data used for sample descriptions.
